# SUSTAINABILITY OF OLDER ADULT VOLUNTEERS IN A MEDICAL EDUCATION PROGRAM

**DOI:** 10.1093/geroni/igae098.1636

**Published:** 2024-12-31

**Authors:** Keri Christensen, Janice Knebl, Jennifer Severance

**Affiliations:** University of North Texas Health Science Center, Fort Worth, Texas, United States; University of North Texas Health Science Center, Fort Worth, Texas, United States; University of North Texas Health Science Center, Fort Worth, Texas, United States

## Abstract

Returning to pre-COVID learning expectations in a post-COIVD learning environment has presented new challenges in medical education. One is the sustainability of older adult volunteers, who are the center piece of a medical education program. Creating a sustainable volunteer program through engagement opportunities provides a space for volunteers to build community. UNTHSC has developed educational opportunities for older adult volunteers to learn about geriatrics as well as to create a space to connect with other volunteers. In the Seniors Assisting in Geriatric Education (SAGE) Program, teams of interprofessional health professions students are paired with volunteer older adults in the community, called Senior Mentors. The students visit their Senior Mentor at home, providing health screenings, safety assessments, community resources, and health education. With the onset of COVID-19 and the transition to virtual meetings with students, volunteers’ attrition was a challenge to administering the program. In response, the SAGE mentors were polled about events they would be interested in attending to increase volunteer engagement. A total of 166 surveys were administered and 78 volunteers responded. Results indicated that the majority (81%) would be willing to attend events and that events would be primarily educational health topics related to older adults. The results of that survey were then used to develop education events held twice a year to give the SAGE volunteers the opportunity to connect to other mentors, to expand their knowledge in caring and advocating for themselves, and to build personal relationships with the team involved with SAGE itself.

